# The ‘forgotten’ lateral patellofemoral ligament: The known unknown

**DOI:** 10.1002/jeo2.12109

**Published:** 2024-08-14

**Authors:** Angelo V. Vasiliadis, Theodorakys Marín Fermín, Emmanouil Papakostas

**Affiliations:** ^1^ Department of Orthopaedic Surgery, Sports Trauma Unit St. Luke's Hospital Panorama Greece; ^2^ Department of Physical Education and Sports Sciences at Serres Aristotle University of Thessaloniki Thessaloniki Greece; ^3^ Centro Médico Profesional Las Mercedes Caracas Venezuela; ^4^ Aspetar Orthopaedic and Sports Medicine Hospital Doha Qatar

## Abstract

Level V.

## Graphical abstract

The lateral patellofemoral ligament (LPFL) is an anatomical structure in the second layer of the knee joint and a stabilizer of the patella against medial dislocation. It has firm attachments to the lateral femoral epicondyle and the lateral edge of the patella and patellar tendon. LPFL is non‐isometric and decreases its length during knee flexion. Most LPFL injuries are iatrogenic (>90%), with LPFL reconstruction being the gold standard treatment option.

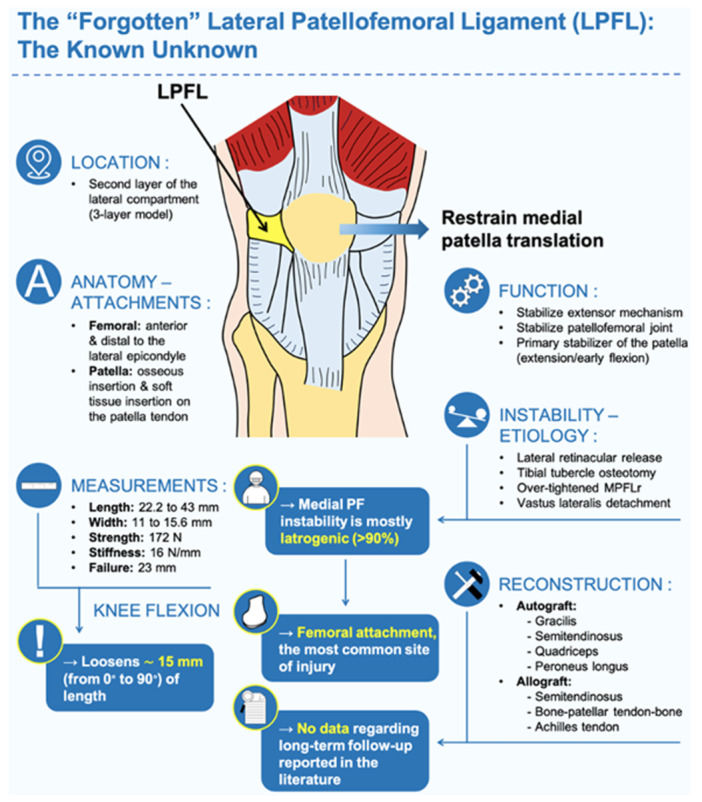

**Abbreviations**
LPFL: lateral patellofemoral ligament
LPFLr: lateral patellofemoral ligament reconstruction
mm: millimetre
MPFL: medial patellofemoral ligament
N/mm: Newton/millimetre
N: Newton

The patellofemoral joint is a unique and complex joint that contains anatomical key structures, playing a crucial role in the balance of the joint [3]. Remarkably, the medial patellofemoral ligament (MPFL) plays an essential role in resisting lateral migration of the patella and keeping the patella within the femoral trochlea [1, 3]. This structure has been well described in the literature [3, 7]. However, the lateral patellofemoral ligament (LPFL) (Figure 1), a primary medial stabilizer of the patella in extension and early flexion [5], remains poorly studied and understood [3, 7].

**Figure 1:**
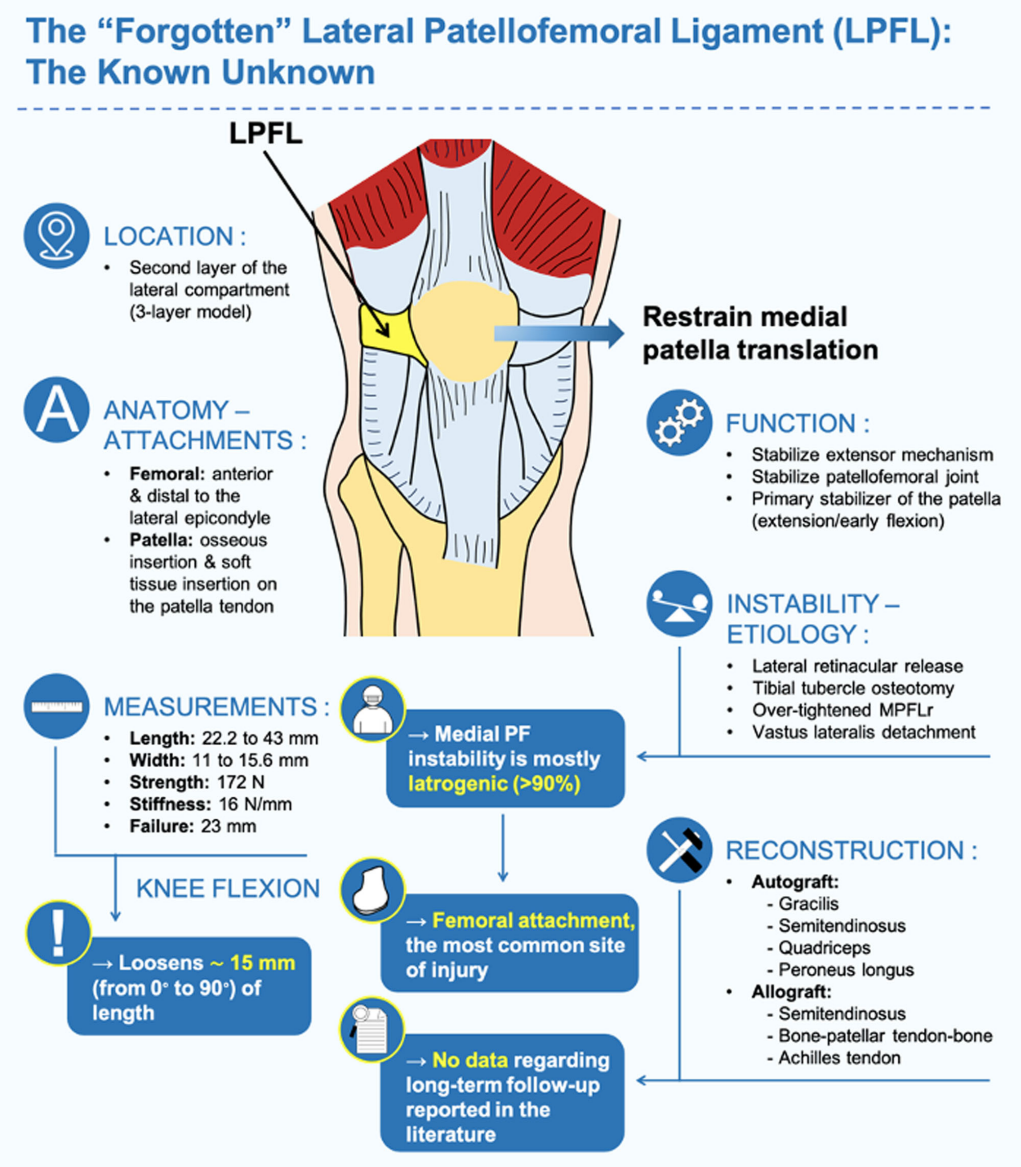
The ‘forgotten’ lateral patellofemoral ligament: The known unknown.

Anatomical studies have shown that the native location of the LPFL is in the second layer of a three‐layer model, between the lateral epicondyle and the patella, within the anterolateral aspect of the knee [1, 5]. The LPFL originates from an osseous area, on average, 10.8 mm anterior and 2.6 mm distal to the lateral femoral epicondyle [4, 7]. At the same time, it has a broad patellar osseous insertion and a soft tissue insertion on the patellar tendon [4]. The mean LPFL length in full extension ranges from 23.2 to 43 mm [1, 3], while the mean LPFL width ranges from 11.7 to 15.6 mm, with femoral attachment being slightly wider when the length of the LPFL increases [3, 7]. Interestingly, LPFL loosens approximately 15 mm when going from 0° to 90° of flexion [4], with greater changes observed early in flexion (from 0° to 30°) [5]. Studies have shown that the LPFL has a mean tensile strength of 172 N and a stiffness of 16 N/mm at 23 mm of displacement, with the femoral attachment being the more prone site of injury [6]. In the literature, medial patellar instability with medial subluxation of the patella is commonly described as an iatrogenic complication (>90%) [7], mainly due to lateral retinacular release [5], followed by overcorrection with medializing tibial tuberosity osteotomy, over‐tightened MPFL reconstruction and detachment of vastus lateralis from the patella [2]. To date, LPFL reconstruction (LPFLr) with different graft sources (autografts and allografts) has been considered the treatment of choice [2, 4]. However, large prospective studies assessing functional outcomes after LPFLr are needed.

## CONFLICT OF INTEREST STATEMENT

The authors declare no conflict of interest.

## ETHICS STATEMENT

Ethics approval and consent to participate and consent for publication are not applicable to this study.
